# Aβ(M1–40) and Wild-Type Aβ40 Self-Assemble into Oligomers with Distinct Quaternary Structures

**DOI:** 10.3390/molecules24122242

**Published:** 2019-06-15

**Authors:** Jacob L. Bouchard, Taylor C. Davey, Todd M. Doran

**Affiliations:** 1 Department of Medicinal Chemistry, University of Minnesota, Minneapolis, MN 55455, USA; bouch171@umn.edu (J.L.B.); davey037@umn.edu (T.C.D.); 2 Institute for Translational Neuroscience, University of Minnesota, Minneapolis, MN 55455, USA

**Keywords:** Alzheimer’s disease, Amyloid-β, recombinant Aβ, oligomers, low-molecular weight (LMW) oligomers, high-molecular weight (HMW) oligomers, quaternary structure, morphology, conformation, PICUP

## Abstract

Amyloid-β oligomers (AβOs) self-assemble into polymorphic species with diverse biological activities that are implicated causally to Alzheimer’s disease (AD). Synaptotoxicity of AβO species is dependent on their quaternary structure, however, low-abundance and environmental sensitivity of AβOs in vivo have impeded a thorough assessment of structure–function relationships. We developed a simple biochemical assay to quantify the relative abundance and morphology of cross-linked AβOs. We compared oligomers derived from synthetic Aβ40 (wild-type (WT) Aβ40) and a recombinant source, called Aβ(M1–40). Both peptides assemble into oligomers with common sizes and morphology, however, the predominant quaternary structures of Aβ(M1–40) oligomeric states were more diverse in terms of dispersity and morphology. We identified self-assembly conditions that stabilize high-molecular weight oligomers of Aβ(M1–40) with apparent molecular weights greater than 36 kDa. Given that mixtures of AβOs derived from both peptides have been shown to be potent neurotoxins that disrupt long-term potentiation, we anticipate that the diverse quaternary structures reported for Aβ(M1–40) oligomers using the assays reported here will facilitate research efforts aimed at isolating and identifying common toxic species that contribute to synaptic dysfunction.

## 1. Introduction

Self-association of the amyloid-β peptide (Aβ) into multimeric oligomers has been linked causally to the progression of Alzheimer’s disease (AD)-related dementia. Aβ oligomers (AβOs) are potent synaptotoxins that inhibit long-term potentiation [1,2,3,4,5,6,7,8,9,10,11] despite comprising only a minority of the aggregated Aβ pool in the brain. AβO intermediates exhibit a variety of biological activities [3,11,12,13,14], which are hypothesized to underlie the nonlinear correlation of Aβ plaque pathology to patient symptomology [15]. Diverse biological functions for AβOs has been attributed to the multitude of quaternary structures they adopt in vitro and in vivo [3,4,10,11,14,16,17,18]. However, defining these important structure–function relationships has been impeded largely due to a lack of methods to control and isolate specific AβO quaternary structures.

A major goal in efforts to understand mechanisms of synaptic dysfunction caused by endogenous AβOs is the preparation of AβOs in vitro that are structurally and functionally similar to their in vivo counterparts. However, AβOs self-assemble via a dynamic equilibrium process, which is characterized by unstable intermediates that interconvert and structurally rearrange upon isolation. Self-assembly is sensitive to environmental conditions [19,20], and experimental handling influences the relative abundance of different oligomeric intermediates [21,22,23]. Therefore, the structures of biologically active AβO assemblies cannot easily be separated for structural characterization, and conditions used to generate different AβO intermediates are difficult to reproduce between laboratories. Therefore, robust methods for generating and analyzing AβO structure–function relationships would accelerate mechanistic studies towards understanding AβO toxicity.

The recombinant Aβ expression system developed by Walsh and coworkers represents a high-yielding source of Aβ that can be produced quickly and in high purity [24,25,26]. The peptide sequence is called Aβ(M1–40) due to the presence of an N-terminal methionine introduced at a start codon. Aβ(M1–40) exhibits fibril aggregation rates that are kinetically faster than synthetically prepared wild-type Aβ40 (herein referred to as WT Aβ40). Nevertheless, oligomers derived from both Aβ(M1–40) and WT Aβ40 potently disrupt long-term potentiation [16]. Structurally, the conformation of di-tyrosine cross-linked Aβ(M1–40) dimers differ from both WT Aβ40 and oxidized Aβ(M1–40)S26C dimers and form different aggregated end-products. These data suggest that their shared synaptotoxicity may be a function of a common aggregation intermediate that features shared quaternary structural elements. Given the importance of endogenous Aβ40 dimers in disrupting synaptic dysfunction [12,16,27], the Aβ(M1–40) monomer might also adopt quaternary structures with biological activity that mirror native AβOs in vivo.

Here, we asked whether common oligomer quaternary structures were shared among intermediates derived from the self-assembly of monomeric WT Aβ40 and Aβ(M1–40). We cross-linked oligomer samples after they were self-assembled to stabilize their native morphologies. We developed a simple assay to characterize the morphology of low-molecular weight (LMW, 8–36 kDa) and high-molecular weight (HMW, 36–250 kDa) species. Under our assay conditions, WT Aβ40 self-assembled into 2mer to 5mer oligomers and Aβ(M1–40) oligomers assembled into a polydisperse mixture of LMW and HMW oligomers. The predominant morphologies of AβOs derived from the two peptides were also distinct, and common intermediates were only present at low concentrations. Using this assay, we rapidly identified oligomer self-assembly conditions that favored HMW Aβ(M1–40) oligomers. Taken together, our results provide evidence that the major oligomeric products of Aβ(M1–40) self-assembly are morphologically dissimilar from those generated by the self-assembly of WT Aβ40, though self-assembly conditions, such as the addition of biomimetic amphiphiles, significantly alter the relative abundance of different quaternary structures. We anticipate that the methods we report to characterize AβO morphologies will find general utility in preparing diverse quaternary structure variants that assist in reconciling mechanisms of pathophysiology during AD.

## 2. Results

### 2.1. Expression and Purification of Monomeric Aβ(M1–40)

**Figure 1:**
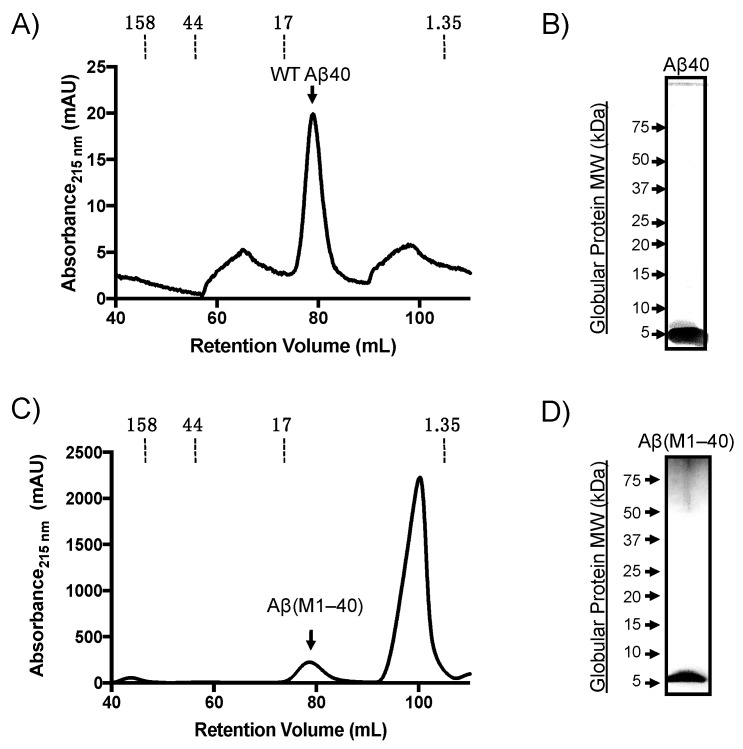
Purification of synthetic and recombinant amyloid-β peptide (Aβ) monomer by size-exclusion chromatography (SEC). (**A**) Representative SEC chromatogram of synthetic wild-type (WT) Aβ40 monomer. (**B**) Silver-stained SDS-PAGE gel of monomeric Aβ40 separated on Criterion™ XT 4–12% bis-tris gradient gels. Globular protein standard molecular weights (left side) are provided for reference. (**C**) SEC chromatogram of Aβ(M1–40) from denatured inclusion bodies following anion-exchange chromatography. (**D**) Silver-stain SDS-PAGE gel of Aβ(M1–40) collected from SEC.

Aβ(M1–40) was expressed using the pET(Aβ1-40) Sac plasmid (provided by D. Walsh) transformed into BL21(DE3)-pLysS competent *E. coli.* Denatured inclusion bodies from expression lysates were purified by anion-exchange followed by gel filtration chromatography as described previously [25]. Synthetic WT Aβ40 was purified on a preparative Superdex HiLoad 16/60 column and eluted with a retention volume of approximately 80 mL (Figure 1A), which was confirmed to be monomeric by SDS-PAGE analysis (Figure 1B). Aβ(M1–40) also eluted at approximately 80 mL retention volume. The peak in Figure 1C labeled as “Aβ(M1–40)” was collected and the presence of a protein with a molecular weight (MW) of approximately 5 kDa was confirmed using SDS-PAGE (Figure 1D). LC-MS characterization indicated that the purity of both WT Aβ and Aβ(M1–40) were approximately 95% (Supplementary Figures S1–S3). Self-assembly studies were therefore carried out without further purification.

### 2.2. WT Aβ40 Oligomers Assemble with Two Distinct Morphologies

**Figure 2:**
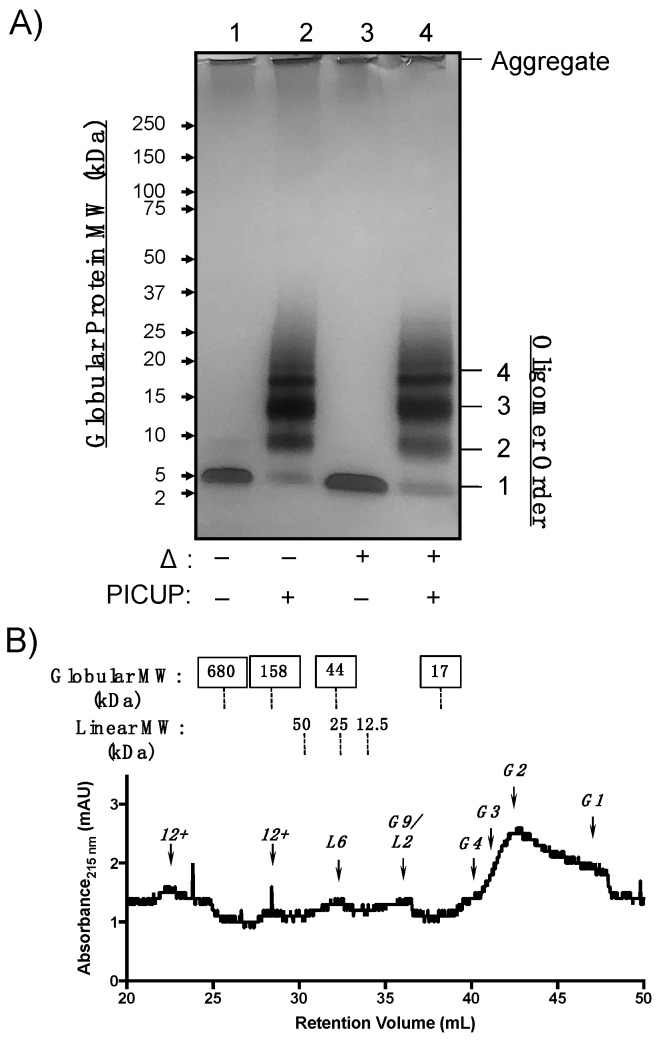
Structural characterization of WT Aβ40 oligomer quaternary structure. (**A**) Silver-stained SDS-PAGE gel of WT Aβ40 oligomers after 24 h incubation at 4 °C in phosphate-buffered saline (PBS). Proteins were separated on Criterion™ XT 4−12% bis-tris gradient gels. Samples in lanes 1 and 3 were not covalently cross-linked. Samples in lanes 2 and 4 were subjected to photo-induced cross-linking of unmodified proteins (PICUP). Lanes 3 and 4 were thermally denatured (Δ) for 5 min at 95 °C prior to SDS-PAGE separation. (**B**) SEC chromatogram of cross-linked WT Aβ40 oligomers separated using three analytical gel filtration columns linked in tandem. Approximate molecular weights of globular (top row, boxed labels) and linear (bottom row) standards are listed above the chromatogram. The oligomer assembly state corresponding to each peak is listed above an arrow for globular (G1, G2, etc.) and linear (L1, L2, etc.) morphologies.

WT Aβ40 oligomers were generated from monomeric peptide diluted to 100 µM in phosphate-buffered saline (PBS) for 24 h. Relative oligomer abundances were analyzed using SDS-PAGE (Figure 2A). Because LMW oligomers are sensitive to SDS-PAGE conditions, oligomer mixtures were cross-linked using photo-induced cross-linking of unmodified proteins (PICUP) [28,29]. PICUP covalently cross-links closely associated heteroaromatic sidechains during photo-irradiation, preventing dissociation of oligomers under denaturing conditions [30,31,32]. WT Aβ40 oligomers that were not subjected to PICUP cross-linking (unmodified WT Aβ40) were sensitive to SDS during PAGE and migrated as monomers and, to a lesser extent, dimers (Figure 2A, lane 1). Cross-linked AβOs migrated as bands corresponding to dimers through pentamer assembly states (referred to as 2mer–5mer). Thermal denaturation (lanes 3 and 4) did not significantly alter the abundance of species that were also observed in lanes 1 and 2. Protein bands were observed in the loading wells that resisted thermal and SDS denaturation, which may be attributed to larger aggregates, such as protofibrils or fibrils with molecular weights (MWs) greater than 250 kDa. These data are consistent with previous WT Aβ40 oligomer cross-linking studies, which found that cross-linked oligomer distributions ranging from 2mer–5mer resolve as monomers by SDS-PAGE in the absence of cross-linking [31,32].

SDS reportedly perturbs AβO assembly states in the absence of covalent cross-linking, so we analyzed cross-linked WT Aβ40 by analytical size-exclusion chromatography (SEC). WT Aβ40 oligomers were cross-linked as described above and separated without denaturation on a tandem gel filtration chromatography system equipped with a Bio-Rad ENrich 650 and two Bio-RAD ENrich 70 columns linked sequentially to improve separation of LMW from HMW AβOs. AβO morphology impacts migration through sepharose columns, resulting in retention volumes that correlate with linear dextran polymer standards [16,17]. Thus, we assigned SEC peaks based on retentions that correlated either to a globular protein or linear dextran MW standard calibration curve (Figures S4–S6). Cross-linked WT Aβ40 oligomeric states migrated predominantly as globular monomers and dimers in the chromatogram shown in Figure 2B. A peak shoulder that eluted at 40 mL corresponding to a globular tetramer was present at low concentrations. However, a distinct globular trimer peak was not observed despite the observation of an intense band corresponding to trimer in the SDS-PAGE gel shown in Figure 2A. AβOs that correlated more closely to linear dextran, or “linear AβOs”, were also observed in low quantities and were retained as putative 2mer, 6mer, and higher-order oligomers (“12+”mers), though no linear trimer was observed. The oligomeric species eluting at 36.5 mL could not be unambiguously assigned, as both calibration curves predicted a globular 9mer or linear 2mer at this retention time. The SEC platform thus complements SDS-PAGE characterization of oligomeric states by detecting morphological differences and low-abundant AβO quaternary structures.

### 2.3. Aβ(M1–40) Oligomers Are More Polydisperse Than WT Aβ40

**Figure 3:**
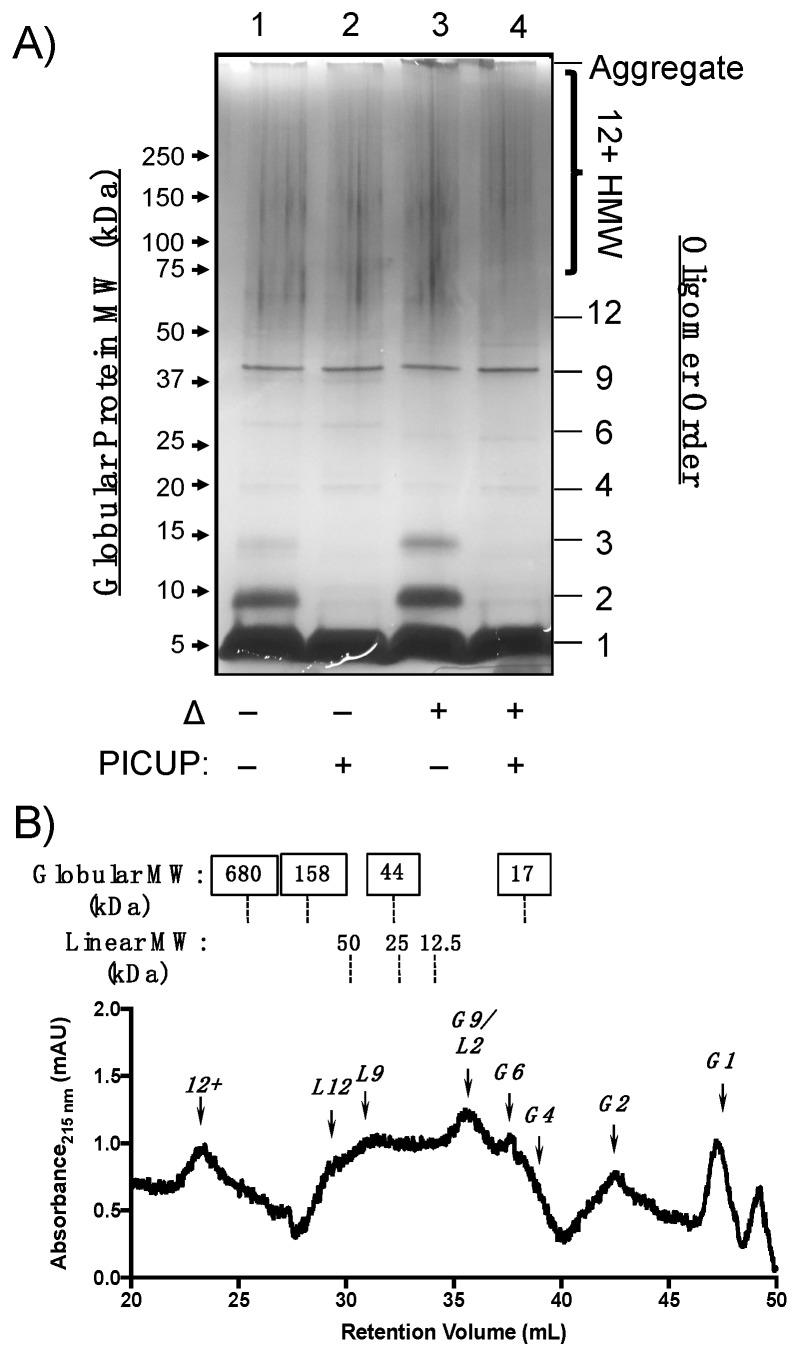
Structural characterization of Aβ(M1–40) oligomer quaternary structure. (**A**) Silver-stained SDS-PAGE gel of Aβ(M1–40) oligomers after 24 h incubation at 4 °C in PBS. Lanes 1 and 3 were not subjected to PICUP crosslinking, whereas lanes 2 and 4 contained cross-linked oligomers. Samples in lanes 3 and 4 were thermally denatured (Δ) at 95 °C for 5 min prior to SDS-PAGE separation. Globular protein standard molecular weights (left side) and oligomer assembly state (right side) are provided for reference. (**B**) SEC chromatogram of cross-linked Aβ(M1–40) oligomers separated on tandem gel filtration columns. Approximate molecular weights of globular (top panel, boxed labels) and linear (bottom panel) standards are listed above the chromatogram. The oligomer assembly state corresponding to each peak is listed above an arrow for globular (G1, G2, etc.) and linear (L1, L2, etc.) morphologies.

Having demonstrated the utility of PICUP cross-linking followed by SDS-PAGE and SEC quaternary structural analyses, we used these methods to characterize Aβ(M1–40) oligomers formed under the same conditions. SDS-PAGE analysis in Figure 3A shows that Aβ(M1–40) assembles into a polydisperse mixture of oligomers, of which only certain species resisted both SDS and thermal denaturation (Figure 3A). For example, SDS treatment denatured unmodified oligomers into 2mers and 3mers (lane 1 versus lane 2), and these bands were more intense after thermal denaturation (lane 1 versus lane 3). Bands in lanes 1 and 2 that migrated with an apparent MW of 25 kDa, shifted to a slightly lower MW upon thermal denaturation (lanes 3 and 4). These changes to both cross-linked and unmodified AβOs observed after thermal denaturation may be due to conformational or morphological changes that influence migration in SDS-PAGE, which are independent of cross-linking.

Protein bands corresponding to HMW oligomers were also observed prominently, regardless of whether the sample was treated with cross-linking reagents. Silver staining artifacts precluded quantification of these species by densitometry, though the low intensity of bands corresponding to dimeric and trimeric species in cross-linked samples suggests that these two species comprise the building blocks of HMW Aβ(M1–40) oligomers. Staining of aggregates in the loading well was less intense for recombinant Aβ(M1–40) than for WT Aβ40 (Figure 2A), indicating that higher order aggregates of Aβ(M1–40) are primarily HMW oligomers after 24 h incubation.

We next used analytical SEC to determine whether Aβ(M1–40) oligomer abundances were consistent under conditions that are not expected to denature quaternary structures. Cross-linked samples containing Aβ(M1–40) oligomers were separated using serial analytical SEC columns and analyzed as described for WT Aβ40. Cross-linked Aβ(M1–40) oligomers migrated as linear and globular morphologies, though relative abundances were different for Aβ(M1–40) oligomers (Figure 3A). Similar to WT Aβ40, globular monomers and 2mers were present in the chromatogram shown in Figure 3B. No trimers were observed in the SEC trace after cross-linking, consistent with the SDS-PAGE analysis. A large peak corresponding to either a globular 9mer or linear 2mer eluted at approximately 36 mL. Given that two bands corresponding to 37 kDa aggregates were detected by SDS-PAGE in Figure 3A, this species is more likely to be a globular 9mer than a linear 2mer.

Globular 6mers and aggregates corresponding to HMW oligomers were abundant in the cross-linked Aβ(M1–40) oligomer SEC trace that were absent in the WT Aβ40 chromatogram. Linear morphologies unique to Aβ(M1–40) eluted as a broad peak between 30–34 min as a mixture of quaternary structures. Distinguishable peaks corresponding to linear 9mers and 12mers were among this mixture. Unfortunately, this region also corresponds to globular HMW AβOs. SDS-PAGE protein bands between 60–150 kDa in Figure 3A are expected to be retained between 29.8 and 34.1 mL in SEC. HMW oligomers with globular and linear morphologies likely co-elute under these SEC conditions, making it difficult to resolve the relative abundance of these HMW AβOs. Therefore, the tandem arrangement of sepharose columns used for SEC in this study cannot adequately resolve certain HMW oligomer quaternary structures.

### 2.4. Aβ(M1–40) Oligomer Folding Pathways Are Sensitive to Micellar Amphiphiles

Given evidence that Aβ(M1–40) oligomer synaptotoxicity results from oligomer intermediates that are larger than 2mers but smaller than their aggregation end-products, we assayed conditions that might be used to favor the generation of large quantities of different oligomeric states. We focused on the use of lipid-mimetic surfactants as additives during self-assembly because they have been shown previously to stabilize or inhibit WT Aβ self-assembly [19,22,23]. As initial validation of the screening approach, we co-incubated Aβ(M1–40) with Tween 20 or SDS above their critical micelle concentration (CMC). Tween 20 has been shown to stabilize pre-formed HMW Aβ42 oligomers, whereas SDS has variable effects on WT Aβ42 self-assembly, depending on the concentration of SDS [22]. Therefore, we initially co-incubated SDS at a concentration of 35 mM, which is above its CMC and is the concentration used in SDS-PAGE loading buffers.

**Figure 4:**
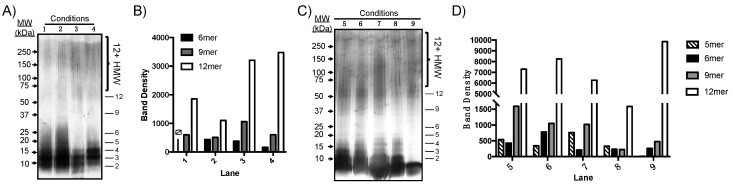
Rapid screening of oligomer growth conditions for Aβ(M1–40) using PICUP and SDS-PAGE analyses. (**A**) Silver-stained SDS-PAGE gel of Aβ(M1–40) oligomers incubated under conditions 1–4. Lane numbers correspond to the conditions listed in Table 1. Proteins were separated on 12% Mini-PROTEAN^®^ TGX™ polyacrylamide gels. (**B**) Graphical representation of oligomer abundance determined using densitometry after 24 h incubation. ND: no 6mers were detected in lane 1. (**C**) Silver-stained 12% TGX polyacrylamide gel of oligomers assembled under conditions 5–9 in Table 1. (**D**) Oligomer abundances from (C) determined using densitometry. Note, the y-axis is segmented due to the intensity of bands corresponding to 12mers.

Aβ(M1–40) was incubated at 100 µM in PBS under various conditions listed in Table 1. After PICUP cross-linking, the resultant oligomers were separated on a 12% polyacrylamide gel, which separates HMW species that were not well-resolved by SEC in Figure 3B. After 24 h, samples were cross-linked using PICUP and oligomer dispersity was analyzed by SDS-PAGE (Figure 4A). A qualitative analysis of silver-stained gels showed that most Aβ(M1–40) exists as lower order aggregates ranging from 2mers to 6mers that were not resolvable using the 12% polyacrylamide gels. However, 9mers to 12mers were resolved clearly enough to measure band intensities using densitometry. Tween 20 had the most significant influence on 12mer and HMW oligomer abundances, even after 24 h incubations (lane 3, Figure 4A,B). Figure 4C shows that after 48 h, oligomers were more polydisperse, though only 5mers–12mers could be resolved for quantification using densitometry. Relative oligomer abundances after 48 h compared to those after 24 h incubations, with the exception of 12mers that were present at levels nearly six-fold higher than any other assembly state, regardless of whether Tween 20 was added. In the absence of PICUP (lane 9), only 12mers were detected in significant quantities when co-incubated with Tween 20. Co-incubation of Aβ(M1–40) with SDS inhibited assembly of 12mers and other HMW oligomers (lane 8). Therefore, co-incubation with Tween 20 has a stabilizing effect on unmodified HMW, but not LMW, oligomers.

Temperature increased the rate of oligomer formation in lane 2 of Figure 4A but did not impact the relative distribution of HMW assembly states, particularly after 48 h incubations. Co-incubation of Aβ(M1–40) monomer with concentrations of SDS used during SDS-PAGE inhibited self-assembly of Aβ(M1–40) oligomers. However, once formed, HMW oligomers are stable to SDS denaturation. Many quantitative analyses of native Aβ in tissue are conducted using SDS-PAGE at room temperature in the presence of detergents such as Tween 20. The results in Figure 4 suggest that native oligomer abundance may be perturbed by these conditions. Cross-linking native AβOs prior to analysis may therefore improve the accuracy of quantitative assays.

## 3. Discussion

Converging evidence implicates specific AβO quaternary structures as pathophysiologically relevant species that induce the synapse loss underlying dementia caused by AD. To unravel AβO structure–activity relationships, AβOs that faithfully model endogenous aggregates must be readily accessible. Recombinant Aβ(M1–40) represents a potential source of AβOs in this regard, as the monomer can be produced in high yields [24,25,26], and oligomers exhibit potent synaptotoxicity. Based on our hypothesis that the LMW oligomers common to both synthetic WT Aβ40 and recombinant Aβ(M1–40) self-assembly pathways contribute to observed synaptic dysfunction, we examined the quaternary structures of oligomers generated from monomers derived from the two peptides under in vitro self-assembly conditions. To overcome the meta-stability of AβOs, they were trapped after 24 h incubation times using PICUP prior to analyses, which enabled characterization of native oligomer orders and morphologies by SDS-PAGE and SEC analyses.

We observed similarities in oligomer size and morphology between WT Aβ40 and Aβ(M1–40) LMW oligomers that may constitute common toxic elements. WT Aβ40 assembled into LMW oligomers ranging from 2mers to 5mers in SDS-PAGE experiments shown in Figure 2A that were sensitive to denaturation by SDS. Aβ(M1–40) assembled into 2mer to HMW oligomers in Figure 3A that could not be easily resolved in silver stained gel. SEC separation revealed that morphologies of WT Aβ40 oligomers are primarily globular, while Aβ(M1–40) oligomers adopted abundant linear and globular morphologies (Figure 2B versus Figure 3B). Linear 6mers and 2mers of WT Aβ40 were also observed in SEC traces (Figure 2B) that were also present in Aβ(M1–40) oligomer samples. However, these shared quaternary structures were detected at low levels in WT Aβ40 samples, suggesting that common toxic quaternary structures, excluding globular dimers, are present at low concentrations.

A major difference between WT Aβ40 and Aβ(M1–40) oligomers was in the abundance of trimers generated from WT Aβ40 after PICUP modification (Figure 2A) that were absent in lanes containing cross-linked Aβ(M1–40) oligomers. SEC traces of oligomers derived from both peptides also lacked trimers, suggesting that WT Aβ40 aggregates comprising these building blocks are unstable under SDS-PAGE conditions. Given that HMW oligomers of WT Aβ40 are present at low concentrations, the cross-linked trimers in SDS-PAGE gels may be derived from dissociated protofibrils arranged into quaternary structures that cannot be cross-linked using PICUP. Conversely, Aβ(M1–40) trimers are stable to SDS-PAGE in the absence of cross-linking (lane 1, Figure 2A), and likely constitute the HMW oligomers (e.g., 6mers, 9mers, 12mers) observed.

WT AβOs assembled with trimer building blocks have been reported as pathophysiologically relevant species with clinical significance [4,7,33]. For example, Lesné and coworkers described an endogenous AβO species in transgenic AD mice, referred to as Aβ*56, that denatured as trimeric building blocks during SDS-PAGE [4,7]. Aβ*56 is synaptotoxic [8,9], despite being present at only a small fraction of the total Aβ pool in AD mouse brain extracts [4,7]. This species differs from other preparations of putative 12mer AβOs, such as globulomers, which dissociate into tetramers and monomers upon denaturation [14]. Aβ*56 and globulomers are derived from the more toxic Aβ42 isoform, which contains two C-terminal amino acids that are absent in Aβ40. Like Aβ(M1–40), some Aβ42 oligomer pathways produce HMW oligomers. Therefore, despite differences in their N- and C-termini, the resultant quaternary structure adopted by Aβ(M1–40) oligomers could potentially model pathologically relevant Aβ42 oligomers.

In an effort to generate abundant quantities of certain oligomeric states derived from Aβ(M1–40), we screened different self-assembly conditions and analyzed the relative abundance of resulting oligomers. PICUP and SDS-PAGE were used to rapidly assess the self-assembly products and their relative distributions. Previous reports describing WT Aβ42 oligomer self-assembly in the presence of amphiphiles showed that Tween 20 stabilizes HMW oligomers once they form. We found this phenomenon to be consistent for Aβ(M1–40) oligomers, as HMW oligomers were detected at higher levels in samples co-incubated in the presence of Tween 20. The use of common biomimetic additives to perturb recombinant AβOs thus generates diverse collections of AβO quaternary structures in a time- and cost-effective fashion.

In addition to identifying preparation conditions for diverse Aβ oligomeric states, the structural characterization methods reported in the present study are expected to assist in LMW and HMW AβO quaternary structure determination. The sensitivity of the SEC platform used in these studies resolved globular and linear LMW AβOs after cross-linking, which enabled us to detect WT Aβ40 oligomers with linear morphologies that were not clearly visualized using SDS-PAGE analysis with silver staining (Figure 2A versus Figure 2B). Cross-linked oligomer distributions determined using SDS-PAGE generally reflected their dispersity observed in non-denaturing SEC analyses. Although, a caveat is that some higher-order oligomers do not cross-link efficiently using PICUP and denature under SDS-PAGE conditions. Therefore, oligomer order and building block composition determined using non-denaturing SEC analyses are an ideal complement to SDS-PAGE when assigning quaternary structures.

Our results also highlight the difficulty in resolving oligomers of similar molecular weight ranges using a single polyacrylamide matrix. Gradient gels in Figure 1, Figure 2 and Figure 3 were well-suited for separating LMW oligomers, while 10% tris-glycine gels separated intermediate and HMW oligomers more effectively (Figure 4). The use of silver-staining to detect AβOs provided the necessary sensitivity to visualize low-abundant oligomeric species, however, artifacts introduced during the development process precluded quantification of HMW oligomers by densitometry. This is especially important given batch-to-batch variability and environmental sensitivity of AβO self-assembly. Therefore, gel matrices must be chosen judiciously based on the desired AβO being quantified.

Finally, we did not investigate the physicochemical origins underlying the differences in Aβ(M1–40) and WT Aβ40 oligomer assembly. However, it is surprising that the presence of an N-terminal methionine has such a profound influence on oligomer self-assembly, particularly because NMR structures of Aβ40 fibrils consistently show that the N-terminus is flexible and disordered in these aggregated end-products [34,35]. The diversity of Aβ(M1–40) oligomers observed during our analyses support previous studies showing that N-terminal modification of Aβ influences oligomer aggregation. For example, molecular dynamic simulations of Aβ dimers predict that the N-terminus stabilizes a β-hairpin between residues 23–27 [36]. The kinetics of β-hairpin folding have been shown experimentally to be a critical step during early stages of Aβ self-assembly [37,38,39]. Moreover, spectroscopic analyses of N-terminal amino acid dynamics in Aβ consistently show that N-terminal residues are involved in early conformational changes during aggregation [40,41]. N-terminal amino acids also play a role in clinical manifestations of AD. Point mutations in this region, such as D7N and H6R, enhance Aβ aggregation propensity in favor of HMW oligomers and lead to early-onset AD [42]. Point mutations that have a neuroprotective effect, such as A2T, reduce aggregation rates of Aβ40 with this mutation [43]. These previous reports implicate the N-terminal methionine as playing a role in Aβ(M1–40) oligomer self-assembly, though the precise interactions that influence the formation of diverse quaternary structures are likely complex and deserve further study.

## 4. Materials and Methods

General. All reagents and supplies were used as received unless otherwise noted. Part numbers are listed for reagents that may impact reproducibility of oligomer assembly and characterization studies. Aqueous buffers were prepared using deionized water filtered on a Barnstead™ Pacific TII water purification system. All PICUP and SEC experiments were conducted in a cold room at 4 °C using pre-chilled plastic microcentrifuge and pipette tips and with solutions stored at 4 °C.

Protein expression. The pET-Sac-Aβ(M1–40) was a gift from Dominic Walsh (Addgene plasmid # 71876; http://n2t.net/addgene:71876; RRID:Addgene_71876). The plasmid was transformed in BL21(DE3)-pLysS competent *E.coli* (Thermo Fischer, cat. no. C600003) [24]. Agar plates containing 50 μg/mL ampicillin and 34 μg/mL of chloramphenicol were streaked with bacterial glycerol stocks and incubated at 37 °C overnight. The following day, individual bacterial colonies were picked and placed in starter cultures consisting of 50 mL of LB broth (25 g/L, Fisher Scientific, cat. no. BP9723) containing 50 μg/mL ampicillin and 34 μg/mL of chloramphenicol in a 250 mL Erlenmeyer flask. The starter cultures were allowed to grow overnight at 37 °C and were shaken at 125 RPM. The next morning, the optical density (OD) at 600 nm of each starter culture was measured using a NanoDrop UV-vis spectrophotometer. Desired ODs ranged from 1.5–1.8 absorbance units (au). A 5 mL aliquot of each starter culture was added to 500 mL of LB broth containing 50 μg/mL ampicillin and 34 μg/mL of chloramphenicol in a 2.8 L flask. The flasks were placed on a shaker at 37 °C at 225 RPM until the OD reached ~0.6 au, which took approximately 4–6 h. Once the desired OD was reached, expression was induced with isopropyl β-d-1-thiogalactopyranoside (IPTG) diluted into the flask to a final concentration of 0.1 mM. Following induction, the bacteria were incubated until growth plateaued as measured using spectrophotometry. Typical incubation times after induction ranged from 4 to 5 h. Cells were pelleted at 4500× *g* for 12 min at 4 °C in 1 L centrifuge bottles. The resulting pellets were stored in 25 mL of buffer A (25 mM tris–base, pH 8.5 containing 5 mM EDTA) at −80 °C until further use. Following expression in *E. coli*, inclusion bodies containing the Aβ(M1–40) peptide were solubilized in 8 M urea buffer after a series of sonication and pelleting steps as described previously [24]. Briefly, the cell pellets were thawed at 4 °C and sonicated at 40 W in 30 s intervals for a total of 8 min (4 min total sonication). The resulting solution was centrifuged at 39,000× *g* for 12 min at 4 °C. Following centrifugation, the supernatant was decanted, and the resulting pellet was resuspended in 25 mL of buffer A. These sonication and centrifugation steps were repeated for a total of three cycles. Following the third centrifugation, the pellet was resuspended in 15 mL of 8 M urea in buffer A. The suspension was sonicated and centrifuged as previously described for one cycle. Following the final centrifugation step, the supernatant containing the solubilized inclusion bodies was decanted and stored on ice. The remaining pellet was discarded. Aβ was further purified using anion exchange chromatography (DEAE -Sepharose Fast Flow Resin, Sigma, DFF100, St. Louis, MO, USA).

Anion exchange chromatography. Approximately 15 mL of DEAE-Sepharose Fast Flow Resin (Sigma, DFF100) was added to a 60 mL fritted syringe. The resin was equilibrated through 10 successive washes of 30 mL buffer A until the flow-through and buffer A pH were equivalent. The 15 mL inclusion body solution was diluted up to approximately 40 mL with buffer A, added to the equilibrated resin, and allowed to incubate for 30 min. The plunger was used to push the solution through the frit until the liquid reached the top of the settled resin. The remaining resin in the syringe was incubated with 25 mL of buffer A for 5 min with gentle shaking. Following incubation, buffer A was eluted using gravity flow and collected. Again, the resin was not allowed to run dry at any point during this process. These steps of incubation and gravity elution were repeated with a 25 mL low salt wash (25 mM NaCl in buffer A) and four 10 mL high salt washes (50 mM NaCl in buffer A). Each elution was characterized by sodium dodecyl sulfate-polyacrylamide gel electrophoresis (SDS-PAGE) followed by silver staining (see below). The elutions containing Aβ(M1–40) as determined by the appearance of a band at approximately 10 kDa were carried forward for further gel filtration purification.

Size exclusion chromatography (SEC) of denatured inclusion bodies. Following anion-exchange chromatography, fractions containing Aβ(M1–40) were dialyzed in SnakeSkin tubing, 3500 MWCO (Thermo Fisher, cat. no. 88242, Waltham, MA, USA) in 4 L of 50 mM ammonium bicarbonate. The ammonium bicarbonate solution was exchanged three times after 8 h of dialysis. Following dialysis, the samples were lyophilized and reconstituted in 5 mL of disaggregation solution (50 mM tris–base, pH 8.5 containing 7M guanidine HCl and 5 mM EDTA) and incubated overnight at room temperature. Then, 5 mL of the dialyzed sample were injected onto a Bio-RAD NGC SEC system equipped with a GE Superdex HiLoad 16/60 column at 4 °C. The peptides were eluted in 50 mM ammonium bicarbonate, pH = 8.5 at a flow-rate of 0.8 mL/min. Fraction collection was begun after a dead volume of approximately 40 mL and peaks were collected in 2 mL fractions, lyophilized, and stored at −80 °C for future use.

SDS-PAGE analysis using gradient gels: 4–12% Criterion™ XT Bis-Tris Protein Gels (Bio-Rad, cat. no. 3450123). To begin, 16.6 µL of 4X XT Sample Buffer containing 10% 2-mercaptoethanol (Bio-Rad, cat. no. 1610791) was added to 50 µL of each sample in a 0.5 mL microcentrifuge tube. Samples requiring thermal denaturation were heated to 95 °C for 5 min in a heating block (ThermoFisher, cat. no. 88870001). When analyzing non cross-linked peptides, samples were centrifuged at 2000× *g* using a benchtop centrifuge for 10 s immediately prior to gel loading. Then, 40 µL of supernatant was loaded into the 45 uL loading well. Gel electrophoresis was performed within a Criterion Cell™ (Bio-Rad, cat. no. 1656001) at 150 V for 60 min using XT MES running buffer (Bio-Rad, cat. no. 161-0789, Hercules, CA, USA). Gels were developed using a Pierce Silver Stain Kit (Thermo Fisher, cat. no. 24612) using the manufacturer’s recommended protocol.

WT Aβ40 monomer preparation. Aβ40 was obtained commercially as a film evaporated from a solution of hexafluoro-2-propanol (HFIP) (GenScript, cat. no. RP1004, Piscataway, NJ, USA). Upon receipt, the peptide was reconstituted in 1 mL HFIP, aliquoted into 100 µg fractions, freeze-dried, and stored at −80 °C before use. Thawed aliquots were reconstituted in DMSO to a concentration of 2 mM and then diluted to desired working concentrations using an appropriate buffer.

WT Aβ40 and Aβ(M1–40) oligomer incubations. To being, 100 µg of either WT Aβ40 or Aβ(M1–40) that had been aliquoted and stored at −80 °C were thawed on ice. While the peptides thawed, detergent stock solutions containing 2% Tween 20 and 20% SDS were prepared in phosphate-buffered saline (PBS). The peptides were diluted in PBS to the desired working concentration and split into 18 µL aliquots in 0.5 mL tubes (Fisher Scientific basix, cat. no. 02-682-000, Waltham, MA, USA). Then, 1 µL of PBS or the appropriate detergent stock solution was added to the peptide solution and the tubes were incubated for 24–48 h at 4 °C.

Oligomer photo-induced cross-linking of unmodified proteins (PICUP). Solutions of 1 mM Tris(2,2’-bipyridy)dichloro-ruthenium (II) hexahydrate (Ru(bpy)_3_Cl_2_; Sigma Aldrich, 544981) and 20 mM ammonium persulfate (APS, Affymetrix, cat. no. 76322, Santa Clara, CA, USA) were prepared in PBS in 0.5 mL microcentrifuge tubes (0.5 mL, Fisher Scientific basix, cat. no. 02-682-000). Tubes were wrapped in aluminum foil prior to the experiment. Notably, APS was prepared fresh daily. To each tube, 1 µL of 1 mM Ru(bpy)_3_Cl_2_ was added followed by 1 µL of 20 mM APS. The sample was mixed thoroughly by pipetting the solution ten times using a 10 uL micropipette. Following mixing, the solution was irradiated with a MagLite™ flashlight light source for 10 s with manual rotation of the microcentrifuge tube around the beam of light. Following light exposure, the reaction was quenched with 8 µL of 3X Laemmli SDS sample buffer containing 20 mM of DTT (Fisher Scientific, cat. no. CX25040) if the samples were to be analyzed by SDS-PAGE. Samples analyzed using analytical size-exclusion chromatography (see below) were quenched with 8 µL of 20 mM DTT in PBS. Following oxidative cross-linking, the tubes were stored at −20 °C pending purification via SEC.

Analytical size exclusion chromatography (SEC) of cross-linked oligomers. SEC was performed on an NGC Bio-RAD equipped with one Bio-RAD ENrich 650 (cat. no. 780-1650) and two Bio-RAD ENrich 70 columns (cat. no. 780-1070) linked in series. Each 20 µL sample containing 100 µM Aβ was loaded onto the column and oligomers were eluted in PBS at 0.3 mL/min. The desired peaks were collected in 2 mL fractions after a dead volume of approximately 30 mL, pooled, and lyophilized.

SDS-PAGE separation in 12% polyacrylamide gels for screening oligomer growth conditions. Protein mixtures were separated via SDS-PAGE using 12% Mini-PROTEAN^®^ TGX™ polyacrylamide gels (Bio-Rad, cat. no. 4561044). Then, 10 µL of 6X Laemmli SDS sample buffer containing 20 mM dithiothreitol (Alfa Aesar, cat. no. J60660, Haverhill, MA, USA) was added to 50 µL of each sample. The protein mixture was thermally denatured by heating the sample in a 0.5 mL microcentrifuge tube at 95 °C for 5 min immediately prior to gel loading. A 43 uL aliquot of sample containing loading buffer was loaded into the 50 µL gel loading well. Gel electrophoresis was performed within a Bio-Rad Mini-PROTEAN^®^ Tetra Cell (Bio-Rad, cat. no. 1658004) at 200 V for 25 min in a TGX running buffer (25 mM tris-base, 192 mM glycine, and 0.1% SDS, pH = 8.3). Protein bands were visualized using silver staining, and band density was measured using ImageJ analysis software [44,45].

LC-MS analysis of WT Aβ40 and Aβ(M1–40). Prior to LC-MS analysis, peptides were desalted using Pierce™ C-18 Spin Columns (Thermo Fisher, cat. no. 89870) using the manufacturer’s protocol. Samples were concentrated using a freeze-dryer. Approximately 20 μg of each peptide was dissolved in 40 μL of 20% acetonitrile (ACN) containing 0.1% formic acid. Then, 8 μL of each solution was injected onto a Thermo Scientific UltiMate 3000 UHPLC equipped with an Agilent Zorbax 3000SB-C3 column (0.3 × 100 mm) connected to a Thermo Scientific LTQ XL™ Linear Ion Trap Mass Spectrometer. HPLC grade ACN and deionized water, each containing 0.1% formic acid, were used as the mobile phase. The sample was eluted at 15 μL/min with a 10–90% ACN gradient over 23 min, at 40 °C. Chromatograms were recorded by measuring absorbance at 215 nm.

MALDI-TOF mass spectrometry analysis of WT Aβ40 and Aβ(M1–40). Prior to MALDI analysis, peptides were desalted using Pierce™ C-18 Spin Columns (Thermo Fisher, cat. no. 89870) using the manufacturer’s recommended protocol. Then, 1 µL of each eluted solution was co-spotted onto a MALDI-TOF sample analysis plate with 1 μL of Super-DHB (2,5-dihydroxybenzoic acid, Sigma Aldrich cat. no. 50862-1G-F) matrix. All analyses were performed using the positive reflector mode collected over a mass range of 1000–6000 Da.

## 5. Conclusions

A detailed mechanistic understanding of AβO-mediated neurotoxicity remains unresolved, and mounting evidence suggests that AβO toxicity is dependent on the quaternary structure of individual component oligomers. Accessing AβOs that morphologically and functionally model endogenous AβOs is expected to facilitate efforts towards unraveling disease mechanisms and targeting toxic species therapeutically. As a step in this direction, we showed that the quaternary structure of oligomers generated with recombinant Aβ(M1–40) are polydisperse and morphologically diverse, adopting both globular and linear morphologies. Conversely, the morphologies of WT Aβ40 oligomers are primarily globular, though linear aggregates are observed at low concentrations. We also identified conditions that stabilize HMW Aβ(M1–40) oligomers, which we expect will provide researchers with accessible biomimetic AβO quaternary structure isoforms that model synthetic or brain-derived oligomers for investigating the biological activity of AβO species. Taken together, our results demonstrate that Aβ(M1–40) and WT Aβ40 oligomers share common quaternary structures, though Aβ(M1–40) oligomers are more structurally diverse. These structural similarities and differences deserve further functional characterization to identify relationships between quaternary structure, biological activity and their clinical significance.

## Figures and Tables

**Table 1:** Aβ(M1–40) oligomer incubation conditions.

Condition ^a^	Incubation Time (h)	Temperature (°C)	Co-Incubation Additive ^b^	PICUP
1	24	4 °C	NA	+
2	24	RT	NA	+
3	24	4 °C	Tween 20	+
4	24	4 °C	SDS	+
5	48	4 °C	NA	+
6	48	RT	NA	+
7	48	4 °C	Tween 20	+
8	48	4 °C	SDS	+
9	48	4 °C	Tween 20	-

^a^ Conditions correspond to SDS-PAGE lane in Figure 4. ^b^ 35 mM (1%) SDS or 0.8 mM (0.1%) Tween 20; NA: No additive denotes samples incubated without detergent.

## Supplementary Materials

The following are available online at https://www.mdpi.com/1420-3049/24/12/2242/s1, Figure S1: MALDI-TOF MS spectra, Figures S2 and S3: Peptide LC-MS chromatograms, Figure S4: Preparative SEC globular molecular weight standard chromatogram, Figure S5: Analytical SEC globular molecular weight standard chromatogram and calibration curve, Figure S6: Analytical SEC linear dextran molecular weight standard chromatogram SEC calibration curves.
