# Supplementary material for: Aβ(M1–40) and Wild-Type Aβ40 Self-Assemble into Oligomers with Distinct Quaternary Structures
Source: Molecules. 2019 Jun 15;24(12):2242. doi: 10.3390/molecules24122242 (PMC6631858; doi:10.3390/molecules24122242)
Supplement: Supplementary file 1 [file molecules-24-02242-s001.pdf]

Aβ(M1–40) and Wild-Type Aβ40 Self-Assemble into Oligomers with Distinct Quaternary Structures

Jacob L. Bouchard [1], Taylor C. Davey [1] and Todd M. Doran [1,2,*]

[1] Department of Medicinal Chemistry, University of Minnesota, Minneapolis, MN 55455, USA; bouch171@umn.edu (J.L.B.); davey037@umn.edu (T.C.D.)

[2] Institute for Translational Neuroscience, University of Minnesota, Minneapolis, MN 55455, USA

* Correspondence: doran@umn.edu

SUPPORTING INFORMATION

Contents:

Figure S1. MALDI-TOF mass spectra of WT Aβ40 and Aβ(M1–40) S2
Figure S2. LC-MS analysis of WT Aβ40 .... S3
Figure S3. LC-MS analysis of Aβ(M1–40) S4
Figure S4. Globular protein standards on GE Superdex HiLoad 16/60 column S5
Figure S5. Globular protein calibration curve on tandem analytical SEC columns S6
Figure S6. Linear dextran molecule calibration curve on tandem analytical SEC columns S7 ....

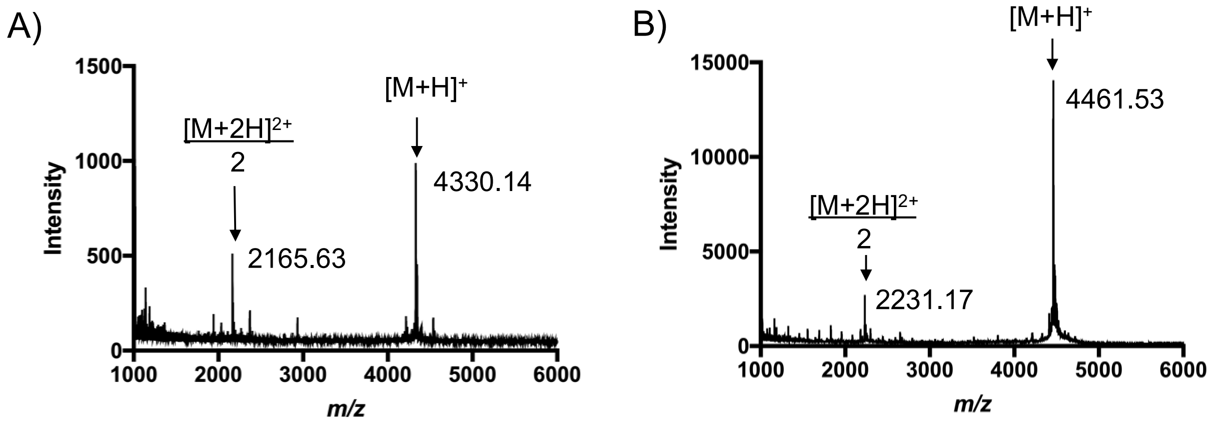

Figure S1. MALDI-TOF spectra of peptides used in this study. **A**) WT Aβ MALDI-TOF mass spectrum. Calculated $[M + H]^+$ = 4330.86 Da; calculated $[M + 2H]^{2+}$ = 2165.93 Da. **B**) MALDI-TOF mass spectra of Aβ(M1–40). Calculated $[M + H]^+$ = 4461.90 Da; calculated $[M + 2H]^{2+}$ = 2131.45 Da.

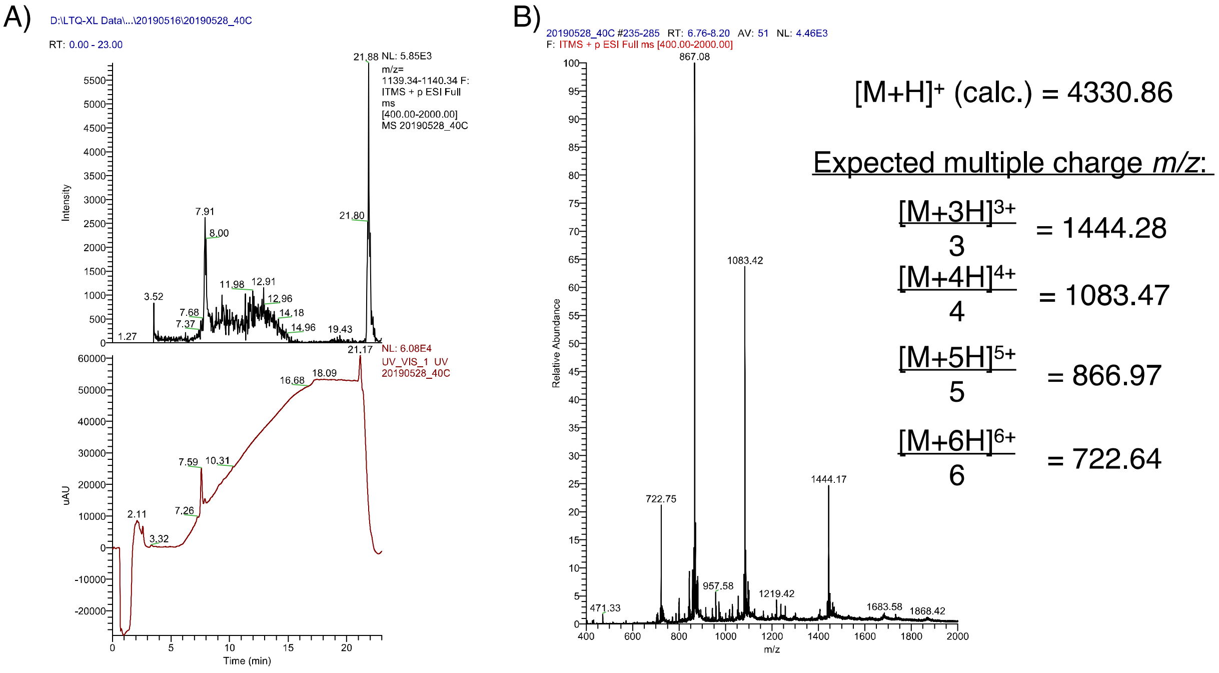

Figure S2. LC-MS analysis of WT Aβ. **A**) Total ion chromatogram (top panel) and UV-Vis absorbance at 215 nm chromatogram (bottom panel). A solvent peak at 2.11 min and a peak at 21 min were in the blank and were therefore not used to determine purity. The chromatographic purity was determined from the LC chromatogram using area under the curve and found to be 95.0%. Figure S2A and determined to be **B**) ESI mass spectrum of sample between 6.76 and 8.20 min of run with calculated m/z values listed on the right.

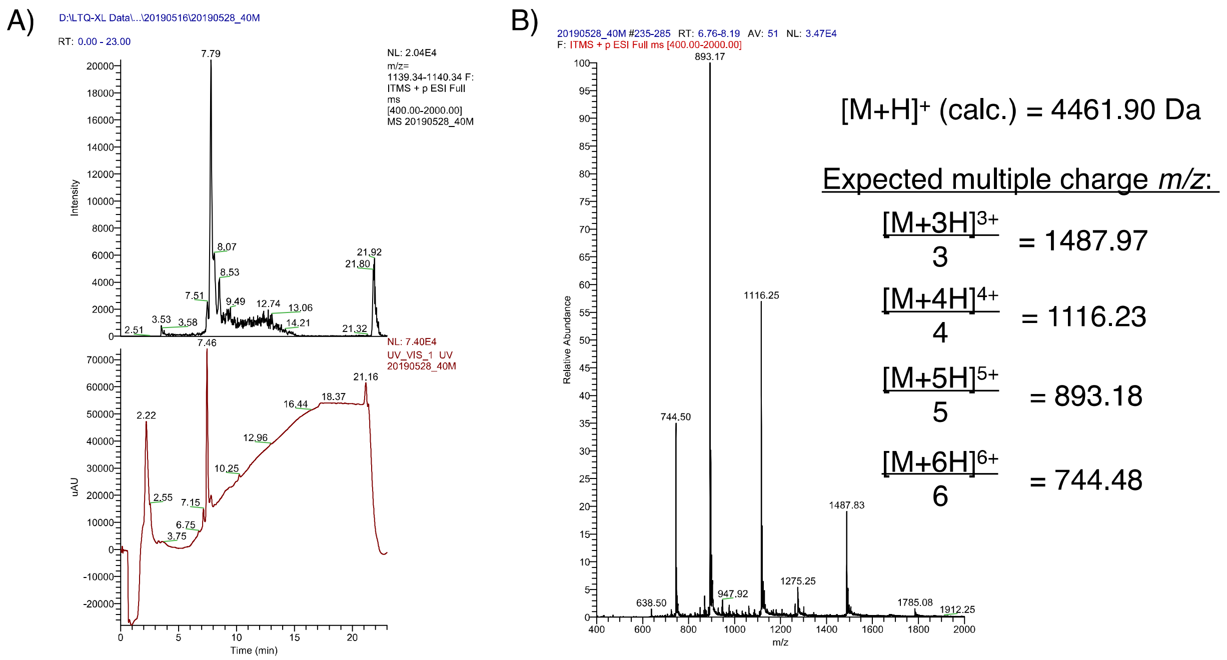

Figure S3. LC-MS analysis of Aβ(M1-40). **A**) Total ion chromatogram (top panel) and UV-vis absorbance at 215 nm chromatogram (bottom panel). Absorbance at 2.2 min corresponded to the solvent peak, and another peak at 21 min was observed in the blank and not used to determine purity. Purity was determined from the LC chromatogram using area under the curve and found to be 94.8%. **B**) ESI mass spectrum of sample between 6.76 and 8.19 min of run with calculated *m*/*z* values listed on the right.

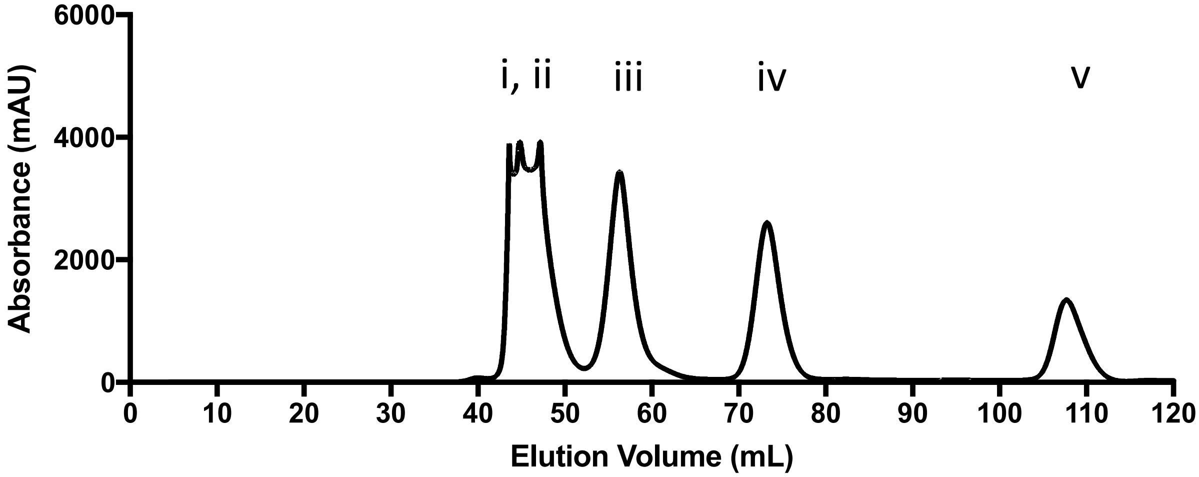

Figure S4. Globular Protein standards on GE Superdex HiLoad 16/60 column. Elution of protein standard calibrants: i) bovine thyroglobulin, 680 kDa; ii) bovine g-globulin, 158 kDa; iii) chicken ovalbumin, 44 kDa; iv) equine myoglobin 17 kDa; v) vitamin B12, 1.35 kDa.

A)

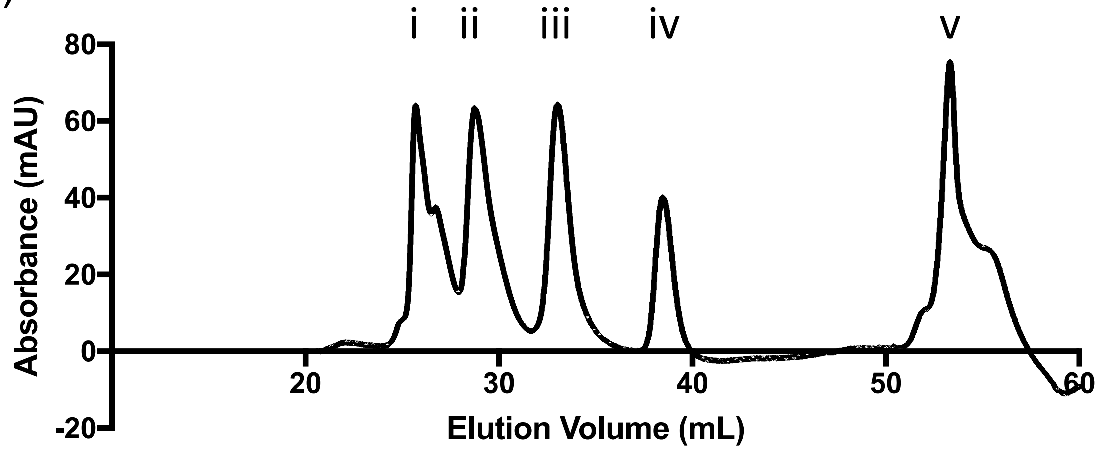

B)

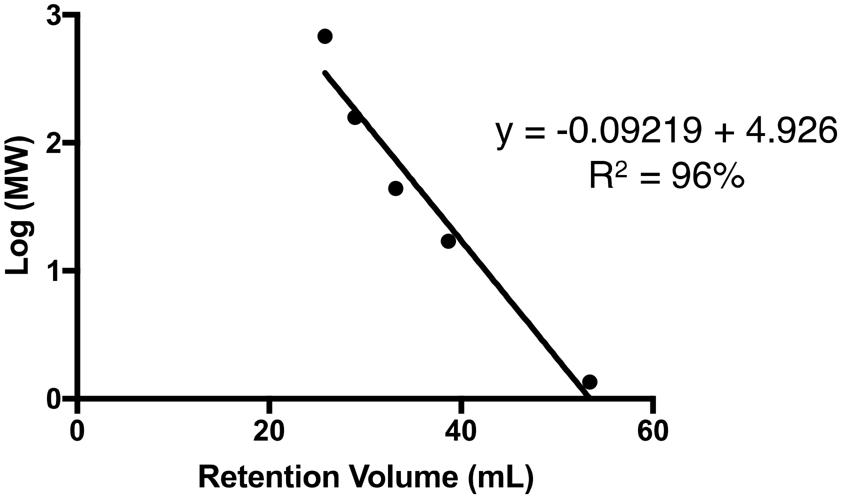

Figure S5. Globular Protein standards using tandem analytical columns (Enrich 650/Enrich70/Enrich70). **A**) SEC chromatogram of protein standard calibrants: i) bovine thyroglobulin, 680 kDa; ii) bovine g-globulin, 158 kDa; iii) chicken ovalbumin, 44 kDa iv) equine myoglobin 17 kDa; v) vitamin B12, 1.35 kDa.; **B**) Calibration curve and linear regression of globular protein standards.

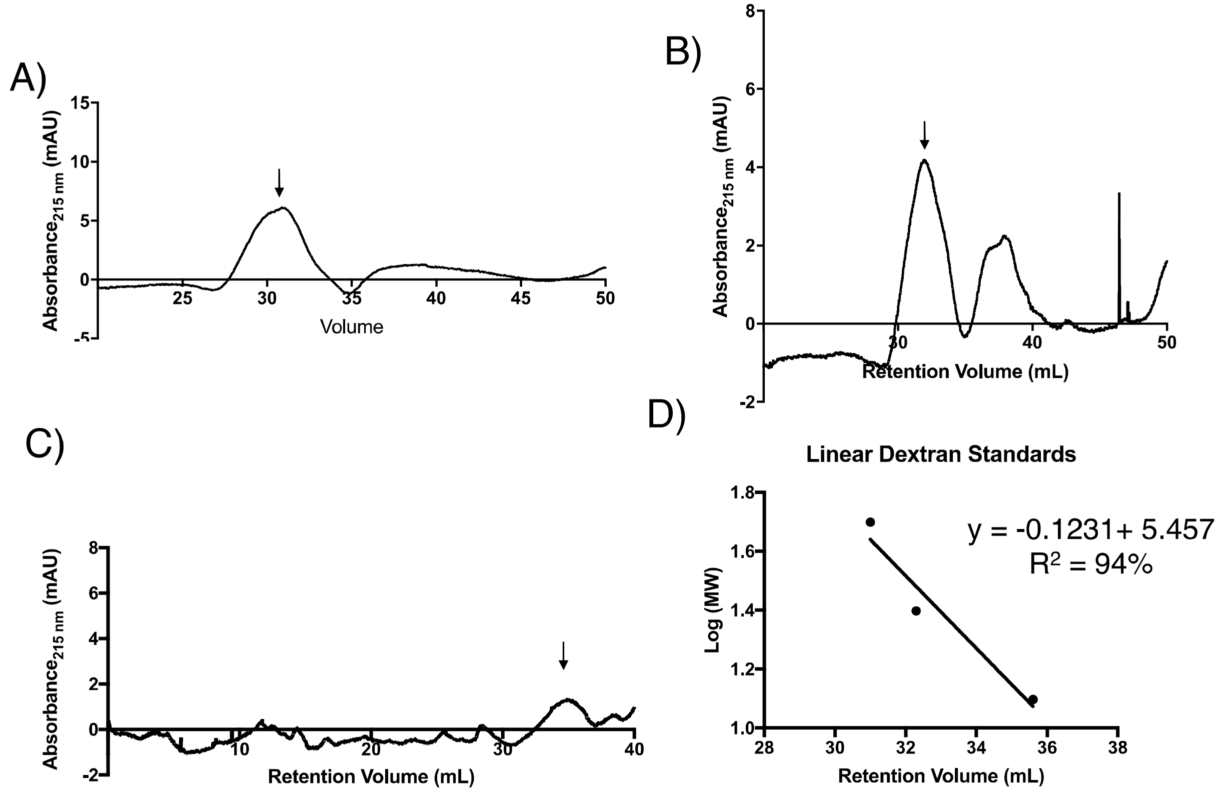

Figure S6. Linear dextran molecule calibration curve on tandem analytical SEC columns. SEC chromatograms with arrows indicating retention time used for calibration curve. **A**) 50 kDa linear dextran standard; **B**) 25 kDa linear dextran standard; and **C**) 12.5 kDa linear dextran standard. **D**) Calibration curve of linear dextran standards.
